# Association of ITGA2 C807T and ITGB3 T1565C polymorphisms with platelet characteristics in hypertensive Azerbaijani patients

**DOI:** 10.21542/gcsp.2025.28

**Published:** 2025-06-30

**Authors:** Vafa Nazirova, Zumrud Ismibayli, Ismayil Gafarov, Faig Guliyev

**Affiliations:** 1 Cardiology Department, XMSK Clinic, Baku, Azerbaijan; 2 Department of Medical and Biological Physics, Azerbaijan Medical University, Baku, Azerbaijan; 3 Cardiology Department, Azerbaijan State Advanced Training Institute for Doctors named after Aziz Aliyev, Baku, Azerbaijan

## Abstract

**Objective:** Cardiovascular diseases (CVDs) are a leading cause of mortality worldwide, including in Azerbaijan. This study investigates the association of *ITGA2* (C807T) and *ITGB3* (T1565C) gene polymorphisms with platelet characteristics in Azerbaijani patients with arterial hypertension (AH), focusing on potential thrombotic risk.

**Methods:** A total of 100 participants were examined, including 76 hypertensive patients and 24 controls. The hypertensive group was further subdivided based on comorbidities: ischemic heart disease (IHD) and type 2 diabetes mellitus (T2DM). Genotyping was performed using the massARRAY method. Platelet indices, including mean platelet volume (MPV), plateletcrit (PCT), and platelet large cell ratio (P-LCR), platelet distribution width (PDWsd) were evaluated. Statistical analysis was conducted using IBM SPSS Statistics 26, with correlation tests, ANOVA, and Chi-square tests to examine genotype distribution and platelet characteristics.

**Results:** Carriers of the homozygous C/C genotype of the *ITGA2* gene and T/T genotype of the *ITGB3* gene exhibited the highest platelet counts and elevated MPV, suggesting a tendency toward increased platelet aggregation. Although all platelet parameters remained within the normal range, significant correlations were found between the *ITGA2* T allele and MPV, PDWsd, and LPCR in hypertensive patients (*p* < 0.05). The study indicates that patients with the T/T genotype of *ITGA2* and ITGB3 may have an elevated risk of thromboembolism.

**Conclusion:** Integrin gene polymorphisms, particularly in *ITGA2* and *ITGB3*, are associated with altered platelet characteristics in Azerbaijani patients with arterial hypertension. These genetic markers could serve as predictors for thrombotic complications, providing insights into targeted prevention strategies in CVD management.

## Introduction

In Azerbaijan, cardiovascular diseases (CVD), particularly coronary heart disease and ischemic heart disease, are a major public health issue, accounting for 85–90% of CVD-related deaths^1,2,3,4^. Recent studies emphasize the role of platelets and their glycoproteins, such as platelet glycoprotein Ia/IIa, in thrombotic and atherosclerotic conditions. Genetic polymorphisms, including those in the PAI-1, ITGA2, and ITGB3 genes, are linked to increased thrombotic risks, such as myocardial infarction and venous thrombosis^5^. Exploring these genetic markers in the Azerbaijani population could improve risk assessment and aid in developing targeted prevention strategies for cardiovascular diseases.

**Aim of the study:** To study the distribution of polymorphic variants of integrin ITGA2 (C807T), ITGB3 (T1565C) and the association of genotypes with platelet characteristics in individuals with arterial hypertension in Azerbaijan.

## Material and Methods

This study was conducted in Baku, Azerbaijan, at the Department of Cardiology of the Special Treatmet Health Complex, laboratory department of Shafa Therapeutic Diagnostic Center, and Department of Cardiology of Azerbaijan State Advanced Training Institute for Doctors named after A. Aliyev. All participants were informed about the study’s purpose and objectives and provided written consent for their involvement. When conducting this research, we adhered to the ethical principles outlined in the World Medical Association’s Declaration of Helsinki, “Recommendations for Physicians Engaged in Biomedical Research Involving Human Subjects”^6^. Ethics committee approval was obtained from Azerbaijan State Advanced Training Institute for Doctors named after A. Aliyev.

The study was conducted between 2019 and 2021, examining a total of 100 patients. The demographic characteristics and clinical data of the study population were presented, dividing them into two groups: case (*n* = 76) and control (*n* = 24). The case group with arterial hypertension (AH) was further divided into three clinical subgroups based on the presence of comorbidities, such as ischemic heart disease and type 2 diabetes mellitus (IHD and T2D): subgroup I, 29 patients with AH; subgroup II, 23 patients with AH and IHD; subgroup III, 24 patients with AH, IHD, and T2D.

The rationale for dividing the study population into subgroups based on the presence of comorbidities was to gain a deeper understanding of how these conditions may impact platelet characteristics and genetic associations. While the small size of the control group may limit statistical significance, we aimed to explore these factors’ contributions to thrombotic risk.

### Inclusion criteria

• Ethnically Azerbaijani patients
• Aged between 30 and 77 years
• Both male and female participants

### Exclusion criteria

• Patients younger than 20 years or older than 77 years
• Pregnant patients
• Patients with congenital heart defects
• Patients with congenital or acquired blood diseases
• Patients undergoing oncology treatments or chemotherapy
• Patients with mental disorders
• Patients with chronic kidney failure

### DNA preparation

DNA was extracted from a small aliquot of whole blood collected in ethylenediaminetetraacetic acid (EDTA) tubes.

Genotyping of selected single nucleotide polymorphisms (SNPs) in the ITGB3 and ITGA2 genes was performed using the MassARRAY^®^ System (Agena Bioscience, USA), which combines matrix-assisted laser desorption/ionization time-of-flight (MALDI-TOF) mass spectrometry with single-base primer extension chemistry for high-throughput, accurate SNP detection.

Target-specific PCR primers were designed using the Assay Design Suite (ADS, Agena Bioscience), flanking the regions of interest for each SNP. The following primer sequences were used for amplification:

ITGB3 (rs5918, PlA1/A2, Leu33Pro)

Forward: 5′-TCTCTGGTGAAAGAGTGTGC-3′

Reverse: 5′-AGTGGGCTGTTGATGTTC-3′

ITGA2 (rs1126643, C807T)

Forward: 5′-GACAGTGACCCTCTGCCTCT-3′

Reverse: 5′-GTTCCCGTGAGGTTCTCTGT-3′

### Statistical analysis

The study was designed as an analytical research project using clinical methods and a scientific sample selection. It was prospective in nature, cross-sectional in duration, and conducted in a clinical setting. Quantitative and qualitative data obtained during the research were analyzed using IBM SPSS Statistics 26, employing variation, discriminant, and dispersion methods. Quantitative indicators are expressed as mean (M, ±SD) and structural indicators (Me, Q1, Q3, min, max). Statistical analyses included Spearman rank correlation. Qualitative indicators are expressed as percentages (%), with comparisons made using Pearson’s Chi-square test. Analysis of variance (ANOVA) was conducted with the F-Fisher test to evaluate the effect of the studied factor on the result, refined by non-parametric methods, taking into account the number of gradations of the factor^7^.

## Results

The mean age of the patients was 50,62 ± 8,55 [32; 69], 58,30 ± 7,59 [41; 77], 59,21 ± 4,62 [48; 76] and 45,87 ± 8,35 [26; 61] in subgroup I, subgroup II, subgroup III and the control group, respectively. Male patients predominated in both case and control groups, accounting for 58.3% and 67.1%, respectively (Table 1).

**Table 1:** Characteristics and clinical data of the study population.

		Control		Main		P*χ*2-value
		Count	N %	Count	N %	
Gender	male	14	58,3%	51	67,1%	0,432
	female	10	41,7%	25	32,9%	
AH	no	24	100,0%	0	0,0%	–
	yes	0	0,0%	76	100,0%	
IHD	no	24	100,0%	29	38,2%	–
	yes	0	0,0%	47	61,8%	
T2D	no	24	100,0%	52	68,4%	–
	yes	0	0,0%	24	31,6%	

**Notes.**

* *p* < 0, 05 is statistically significant.

** The reference genotypes used the chi-square (*χ*^2^) to compare genotypes frequencies.

Allele distribution for the study SNPs differs between cases and controls.

The data presented in Table 2 shows that the difference in the prevalence rate of the normal genotype polymorphism in the T/T homozygous genotype of the ITGB3 gene in AH patients and the control group was not significant (P_H_  =  0,589). As a result of the study, we established the highest incidence of carriage of the homozygous genotype C/C of the ITGA2 gene and homozygous genotype T/T of the ITGB3 gene (Table 2).

**Table 2:** Distribution of genotypes in case and control groups.

Genes/genotypes		Control	Main group	P*χ*^2^-value	P_U_-value
		Count (N %)	Count (N%)		
*ITGB3*	TT – normal genotype	19 (79,2)	56 (73,7)	0,513	0,521
	TC- heterozygous genotype	5 (20,0)	16 (21,1)		
	CC – homozygous genotype	0 (0,0)	4 (5,3)		
*ITGA2*	CC - normal genotype	12 (50,0)	50 (65,8)	0,235	0,117
	CT - heterozygous genotype	6 (25,0)	17 (22,4)		
	TT - homozygous genotype	6 (25,0)	9 (11,8)		

**Notes.**

* *p* < 0, 05 is statistically significant.

The data presented in Table 3 indicate that the highest platelet counts (PLT) were observed in carriers of the C/C normal homozygous genotype of the ITGA2 integrin gene and carriers of the T/C heterozygous genotype of the ITGB3 integrin gene. The mean platelet volume (MPV) was relatively high in patients with the altered homozygous variant genotype of the ITGA2 gene and patients with the altered homozygous ITGB3 genotype. Although the MPV values remained within the normal reference range (7.6−9.0 fl), there was a noticeable tendency towards increased platelet aggregation in these patients. The mean platelet large cell ratio (P-LCR) was higher in patients with the T/T variant homozygous genotype of the ITGA2 gene and in patients with the C/C variant homozygous genotype of the ITGB3 gene.

**Table 3:** Association of *ITGA2, ITGB3* integrin genes polymorphisms with platelet characteristics in the main group of patients.

Gen, genotypes	Statistical parametrs	PLT, 10^3^/μL	MPV, fL	PCT, %	PDW_sd_, fL	LPCR, %
***ITGA2***						
CC	Valid, n	50	49	50	50	48
	M	207,2	8,20	0,16	12,14	18,27
	Me	201,0	8,30	0,17	10,60	17,05
	Q1	175,0	7,70	0,13	10,00	13,80
	Q3	234,0	8,90	0,18	12,30	23,75
CT	Valid, n	17	17	17	17	17
	M	191,9	8,17	0,15	12,48	19,48
	Me	172,0	8,50	0,14	10,50	17,60
	Q1	144,0	8,20	0,12	10,30	16,80
	Q1	230,0	8,80	0,18	13,20	21,33
TT	Valid, n	9	9	9	9	9
	M	188,4	8,66	0,16	11,58	19,59
	Me	193,0	8,70	0,15	11,00	20,60
	Q1	157,0	8,00	0,13	10,00	14,90
	Q3	203,0	9,40	0,18	12,10	24,90
P_H_		0,341	0,494	0,584	0,830	0,576

Gen, genotype	Statistical parameters	PLT, 10^3^/μL	MPV, fL	PCT, %	PDW_sd_, fL	LPCR, %
***ITGB3***						
TT	Valid, n	56	55	56	56	55
	M	195,6	8,27	0,15	12,18	18,82
	Me	188,0	8,50	0,15	10,60	17,50
	Q1	156,0	7,80	0,12	10,55	14,30
	Q3	225,5	8,90	0,18	12,40	24,20
TC	Valid, n	16	16	16	16	15
	M	224,3	8,16	0,18	11,68	17,27
	Me	219,0	8,40	0,18	10,80	17,60
	Q1	184,0	7,70	0,15	9,85	12,60
	Q3	241,5	9,20	0,20	11,90	21,20
CC	Valid, n	4	4	4	4	4
	M	193,3	8,40	0,16	13,55	22,53
	Me	209,5	8,55	0,16	11,35	21,35
	Q1	170,5	7,60	0,14	10,30	16,45
	Q3	216,0	9,20	0,18	16,80	28,61
P_H_		0,155	0,949	0,156	0,688	0,407

**Notes.**

* *p* < 0, 05 is statistically significant.

Importantly, all platelet count parameters remained within the normal range (PLT: 150–400 10^3^/µL, MPV: 8.0–13.0 fL, PDWsd (platelet distribution width in femtoliter):10.0–15.0 fL, PCT: 1.100−1.400%, P-LPCR: 11.0–45.0%)

These results suggest a tendency for increased platelet aggregation in carriers of the altered genotypes of the ITGA2 and ITGB3 genes.

**Figure 1.**
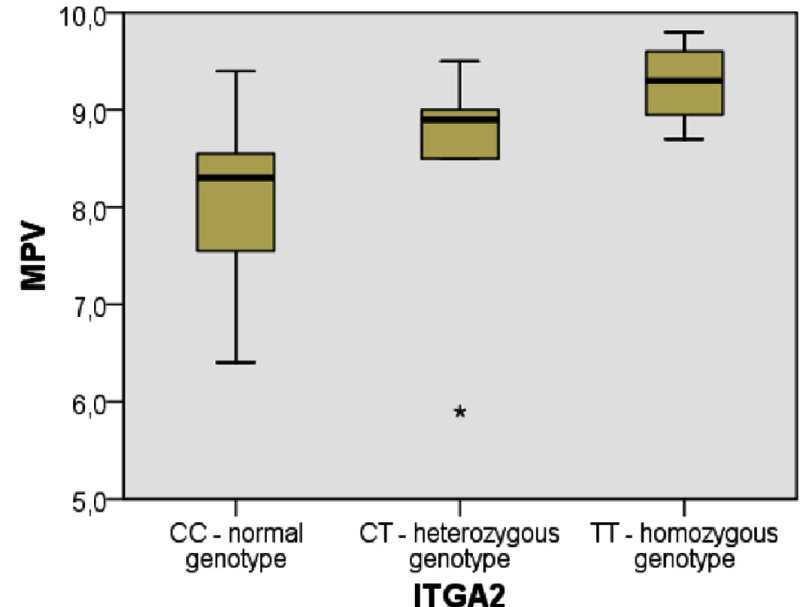
Distribution of MPV value between *ITGA2* gene genotyes.

**Figure 2.**
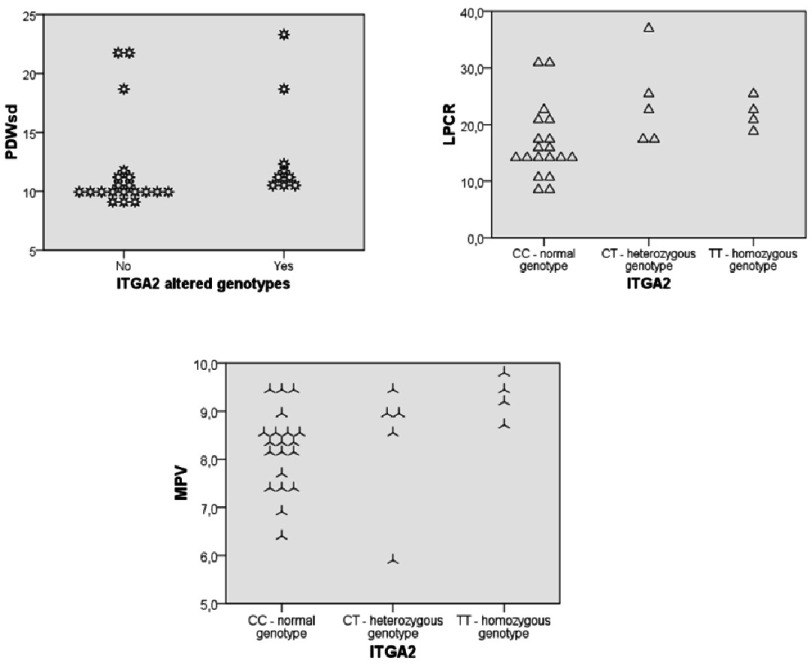
Correlation of platelet indices with *ITGA2* gene.

The highest MPV in the carriers of the altered homozygous T/T genotype in subgroup I (compared to the control index, P_H_  =  0,036) (Figure 1).

We also examined the correlation between the studied genes and thrombocytic indices. In the arterial hypertension (AH) subgroup, a positive correlation was found between the ITGA2 gene T allele with the MPV indicator (Rho  =  0,467; *p* = 0,011), and between ITGA2 altered genotypes, and MPV (Rho  =  0,434; *p* = 0,019), as well as ITGA2 altered genotypes (Rho  =  0,391; *p* = 0,036). The correlation between the ITGA2 gene T allele with the PDWsd indicator (Rho  =  0,427; *p* = 0,021), and with the ITGA2 altered genotypes (Rho  =  0,446; *p* = 0,015) was also positive. Additionally, the ITGA2 gene T allele was positively correlated with the P-LCR indicator (Rho  =  0,525; *p* = 0,004), and ITGA2 altered genotypes (Rho  =  0,535; *p* = 0,003) (Figure 2).

A correlation was also found between the ITGB3 gene C allele, ITGB3 altered genotypes, and ITGA2 gene T allele with P-LCR (Rho  =  1,000) (Table 4).

**Table 4:** Correlation between platelet indices and *ITGA2*  gene genotypes in AH subgroup.

	**ITGA2 T-allele**	**ITGA2 altered genotype**
PLT	*ρ* = − 0.201	*ρ* = − 0.169
	*p* = 0.297	*p* = 0.380
MPV	*ρ* = 0.467^*^	*ρ* = 0.434^*^
	*p* = 0.011	*p* = 0.019
PCT	*ρ* = − 0.089	*ρ* = − 0.063
	*p* = 0.647	*p* = 0.746
PDWsd	*ρ* = 0.427^*^	*ρ* = 0.446^*^
	*p* = 0.021	*p* = 0.015
P-LCR	*ρ* = 0.525^*^	*ρ* = 0.535^*^
	*p* = 0.004	*p* = 0.003

**Notes.**

*ρ* - Spearman’s rho correlation coefficient.

* Correlation is significant at the 0.05 level.

In the AH+ IHD subgroup, an inverse correlation was observed between PLT and the ITGA2 gene, ITGA2 gene T allele (Rho  =  0,433; *p* = 0,039), and ITGA2 polymorphism (Rho  =  0,482; *p* = 0,020) (Table 5).

**Table 5:** Correlation results between platelet indices and  *ITGB3* and *ITGA2* gene genotypes in AH+IHD subgroup.

	**ITGB3**	**ITGA2**	**ITGB3 C-allele**	**ITGA2 T-allele**	**ITGB3 altered genotypes**	**ITGA2 altered genotypes**
PLT	*ρ* = 0.331	*ρ* = − 0.433^*^	*ρ* = 0.331	*ρ* = − 0.433^*^	*ρ* = 0.334	*ρ* = − 0.482^*^
	*p* = 0.122	*p* = 0.039	*p* = 0.122	*p* = 0.039	*p* = 0.120	*p* = 0.020
MPV	*ρ* = − 0.126	*ρ* = 0.018	*ρ* = − 0.126	*ρ* = 0.018	*ρ* = − 0.094	*ρ* = 0.037
	*p* = 0.576	*p* = 0.936	*p* = 0.576	*p* = 0.936	*p* = 0.677	*p* = 0.869
PCT	*ρ* = 0.227	*ρ* = − 0.273	*ρ* = 0.227	*ρ* = − 0.273	*ρ* = 0.247	*ρ* = − 0.318
	*p* = 0.298	*p* = 0.208	*p* = 0.298	*p* = 0.208	*p* = 0.256	*p* = 0.140
PDWsd	*ρ* = 0.061	*ρ* = − 0.239	*ρ* = 0.061	*ρ* = − 0.239	*ρ* = 0.024	*ρ* = − 0.220
	*p* = 0.784	*p* = 0.272	*p* = 0.784	*p* = 0.272	*p* = 0.914	*p* = 0.312
P-LCR	*ρ* = 0.081	*ρ* = − 0.140	*ρ* = 0.081	*ρ* = − 0.140	*ρ* = 0.043	*ρ* = − 0.119
	*p* = 0.719	*p* = 0.534	*p* = 0.719	*p* = 0.534	*p* = 0.850	*p* = 0.597

**Notes.**

*ρ* - Spearman’s rho correlation coefficient.

* Correlation is significant at the 0.05 level.

## Discussion

Analyzing our results in conjunction with existing literature, we note that platelet glycoprotein Ia/IIa, a key member of the integrin family, plays a crucial role in the function of platelet collagen receptors. These glycoproteins act as mediators between cells or between cells and the extracellular matrix, facilitating the adhesion of leukocytes and platelets to the vascular endothelium. This process is essential in both physiological and pathological contexts, including inflammatory and immune responses, as well as in the development of atherosclerosis and thrombosis^8,9^.

Currently, polymorphisms in integrin gene family members, particularly integrin alpha-2 (ITGA2) and integrin beta-3 (ITGB3), have been associated with an increased risk of thrombotic and arterial atherosclerotic diseases^10^. Integrins are adhesion molecules that contribute to platelet aggregation, leading to thrombus formation. Beyond their structural roles, integrins also mediate signal transduction from the extracellular matrix to the cell interior via associated signaling and adapter molecules. This ability to convert mechanical signals into biochemical responses is a critical function of integrins^11,12^. Given that integrin dysregulation is implicated in various diseases, including atherosclerosis and cardiac hypertrophy, considerable efforts have been made to understand how integrins interact with and integrate into other receptor systems.

In our study, platelet indices were evaluated in patients who were carriers of different integrin genotypes. These indices including platelet count, mean platelet volume, platelet size distribution width, thrombocrit, and platelet large cell ratio can serve as promising diagnostic and prognostic markers for thrombotic complications^13^. While the platelet indices differed between the clinical group and the control group, the changes were not statistically significant (p>0,05).

According to our data, the highest MPV levels were observed in patients with AH who were carriers of the T/T altered homozygous genotype of the ITGA2 gene and C/C altered homozygous genotype of ITGB3 gene. Additionally, in patients with AH higher PLT values were observed in carriers of the T/C altered heterozygous genotype of ITGB3 gene, and higher PDWsd and LPCR values were noted in carriers of the T/T altered genotype of ITGA2 gene, although these differences were not statistically significant (p>0,05).

These integrin genes are vital components of biochemical pathways associated with IHD^14^. Our data suggest that using a panel of markers, rather than a single polymorphism, provides a more effective means of predicting thrombotic risk, as the polymorphisms within the panel act in an additive manner. Consequently, genotyping patients based on a panel of hemostasis and coagulation markers is recommended^15–17^.

## Conclusion

The study of allele variations in genes involved in the hemostatic system enables the prediction of diseases linked to hemostatic disorders, such as coronary heart disease and ischemic stroke. This knowledge allows for the timely implementation of preventive measures to improve disease prognosis. Our research has identified key genotypes within the integrin complex that are most responsible for blood clot formation in patients with arterial hypertension. Expanding our understanding of genetic predictors will enhance the identification of risk factors for thromboembolism in cardiovascular pathology.

## Study limitations

This study has several limitations. First, the relatively small sample size reduces the statistical power, which may limit the ability to detect more subtle genetic influences on platelet characteristics. A larger cohort would allow for more robust conclusions regarding the associations between ITGA2 and ITGB3 gene polymorphisms and thrombotic risk.

Additionally, the study focused only on two specific gene polymorphisms. While these integrins play a key role in platelet function, other genetic factors involved in the coagulation pathway and platelet aggregation may also contribute to thrombotic risk. Expanding future research to include additional genetic markers could provide a more comprehensive understanding.

Finally, while platelet indices such as mean platelet volume and platelet large cell ratio were analyzed, other factors like lifestyle, diet, and comorbidities may influence platelet function and were not fully controlled in this study. Considering these variables in future research would offer a more nuanced view of the genetic and environmental interactions affecting thrombotic risk.

## Declaration of competing interest

The authors declare that they have no known competing financial interests or personal relationships that could have appeared to influence the work reported in this paper.
